# Immune-related keratitis is a rare complication associated with nivolumab treatment in a patient with advanced colorectal cancer: A case report

**DOI:** 10.3389/fonc.2022.1021713

**Published:** 2022-11-15

**Authors:** Yuqi Su, Guoquan Li, Jiaxin Xu, Jiale Zheng, Jiapeng Jiao, Jianhui Zhang, Xiaokang Gu, Zhai Cai, Hongyu Luo, Zhou Li, Shuai Han

**Affiliations:** ^1^ The Second School of Clinical Medicine, Southern Medical University, Guangzhou, China; ^2^ Department of General Surgery, Guangdong Province Huizhou Sixth Hospital, Huizhou, China; ^3^ Department of Gastrointestinal Surgery, General Surgery Center, Zhujiang Hospital, Southern Medical University, Guangzhou, China

**Keywords:** immune-related keratitis, corneal ulceration, metastatic colorectal cancer, nivolumab, immune checkpoint inhibitors, matrix metalloproteinases, maspin

## Abstract

**Background:**

Immunotherapy has been widely used to treat Colorectal cancer but has also observe some immune-related adverse effects. With proper treatment, most irAE can be solved and the effect of immunotherapy will not be affected by temporary immunosuppression. However, there are few reports about corneal irAE, and the current understanding of irAE is incomplete. Here we report a metastatic colorectal cancer case of immune-related keratitis caused by nivolumab and to explore the occurrence of immune-related keratitis.

**Case description:**

Here we report the case of a 49-year-old man with mCRC who had no previous ocular disease but developed immune-related ulcerative keratitis after treatment with nivolumab. We summarize a large amount of literature to discuss the mechanism of immune-related keratitis. In addition, we conclude a method that may be used to detect the occurrence of immune keratitis, by monitoring MMPs and maspin in patients treated with nivolumab. We believe immune-related keratitis may be a rare complication of nivolumab in the treatment of mCRC. The effect of simple anti-infective therapy and repair-promoting drugs was not obvious, but the effect of glucocorticoid combined with autologous serum was significant.

**Conclusion:**

The mechanism of immune-related keratitis is that nivolumab destroys the immune microenvironment and ACAID, and affects corneal healing. Patients who use nivolumab can prevent immune keratitis by testing MMPs and maspin. The occurrence of immune keratitis may be a good indicator of the efficacy of ICI, and further study can be done in the follow-up.

## Introduction

Colorectal cancer (CRC) is the third most common cancer worldwide and the second leading cause of cancer death worldwide after lung cancer (1). Sadly, the five-year survival rate of patients with metastatic colorectal cancer (mCRC) is less than 20%. Systemic treatment is considered to be the most important treatment for such patients such as chemotherapy, targeted therapies and immunotherapies (2). Studies have found that mCRC patients can be treated according to the molecular and pathological characteristics of the tumor, which can improve the overall survival rate (2).

Immunotherapy for microsatellite stable (MSS) mCRC patients has been a challenge, as previous studies have found that MSS-type CRC patients do not benefit from immune monotherapy (3). Moreover, MSS-type CRC patients account for 85% of all CRC patients, and the prognosis is worse than that of MSI-H/dMMR-type patients (4, 5). In 2020, a Japanese scholar organized a clinical trial of regorafenib combined with nivolumab in the treatment of gastric cancer and colorectal cancer. The results showed that regorafenib could enhance the antitumor activity of anti-PD-1 immunotherapy and had a manageable safety profile and encouraging antitumor activity in patients with gastric and colorectal cancer (6).

However, with the widespread use of immunotherapy in the clinic, more and more immune-related adverseeffects (irAE) were observed. The main adverse reactions related to anti-PD-1/PD-L1 are rash, colitis, hepatitis, endocrine diseases, and so on. The use of corticosteroids or other immunosuppressants has been commended as effective treatment for irAE. Fortunately, with proper treatment, most irAE can be solved and the effect of immunotherapy will not be affected by temporary immunosuppression (7, 8). However, there are few reports about corneal irAE, and there is no comprehensive understanding of irAE at present (9). Here we report a case of MSS with RAS mutant mCRC who had no previous ocular disease but developed immune-related keratitis after nivolumab treatment.

## Case presentation

**Figure 1:**
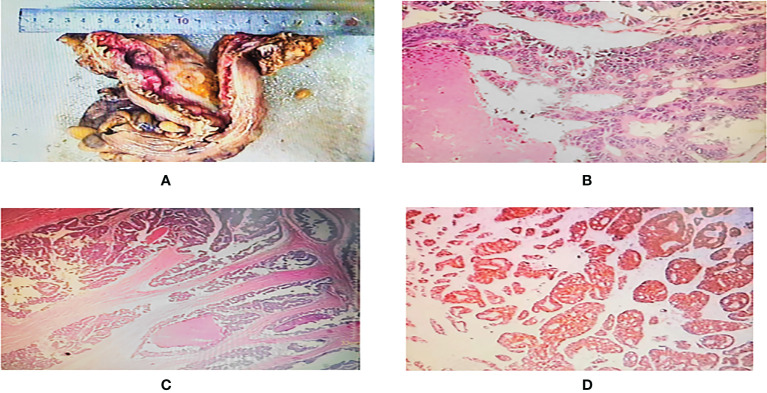
**(A)** One segment of intestinal canal, 21.5 cm long, 7.5-11 cm circumferential diameter, 2.5 cm tangential margin at one end, one ulcerated mass visible at 11.5 cm at the other end, 4.5x4x2.3 cm in size, grayish white cut surface. **(B, C)** Microscopic observation: the tumor cells were arranged in adenoid or sieve shape, infiltrated and broke through the plasma membrane layer, intravascular cancer thrombus and nerve invasion were seen, the tumor cells had abundant red stained cytoplasm, large and deep stained nuclei, obvious nucleoli, easy to see nuclear fission, and focal necrosis. **(D)** Immunohistochemistry showed tumor cells: CK(+), CK7 (–), CK20(+), CDX-2(+), MLH-1(+), MSH2(+), MSH6(+), PMS2(+), P53(+), Ki67 about 90%(+).

A 49-year-old man was admitted to the hospital with ongoing diarrhea in December 2018. The patient underwent colonoscopy and then a biopsy was taken from colon tissue. Endoscopic results showed that a polypoid lesion(1.5×2CM) was observed 6 cm from the anal verge and a cauliflower-like mass with a crisp texture and easy bleeding was observed 18 cm from the anal verge. The pathological examination of the specimen demonstrated moderately differentiated adenocarcinoma in the sigmoid colon and tubulovillous adenocarcinoma with high-grade intraepithelial neoplasia in the rectum. According to abdominal plain computed tomography (CT) scans, there are multiple round low-density shadows with unclear boundaries within the liver parenchyma. Contrast-enhanced CT scans showed that the enhancement degree of lesions in the arterial phase was similar to that of adjacent liver parenchyma, whereas the enhancement degree of lesions in the venous phase and portal phase was lower than that of the liver parenchyma. Therefore, the patient was diagnosed with colorectal cancer with liver metastases. Previously, he had grade 2 hypertension and there was no similar patient in his family. After a comprehensive evaluation, the rectal cancer resection (Dixon surgery) and colostomy were performed and postoperative pathological examination revealed moderately differentiated colorectal adenocarcinoma (pT4bN2M1 stage IVc) (**Figure 1**). According to the results of the molecular pathological examination, mutated KRAS (condon12/13 mutation in exon 2) was found. The tumor microenvironment (TME) of the patient was MSS.

### First-line treatment

From two weeks after the operation, the patient received four cycles of chemotherapy with XELOX (oxaliplatin 130mg/m^2^and capecitabine1000mg/m^2^) every three weeks. Re-examination of MRI suggested that the liver was slightly enlarged with multiple nodules. Besides, the blood routine test indicated that red blood cells, white blood cells and platelets all decreased and myelosuppression caused by chemotherapy was considered. Due to the increase in tumor indexes, the regimen was adjusted to XELOX plus bevacizumab (5mg/Kg) in March 2019 for a total of three cycles. Re-examination of CT showed that the intrahepatic metastases were smaller than before. But unfortunately, multiple small nodules could be observed in the lungs. According to the imaging findings and patient history, we considered it as multiple metastases in the lungs. One month after the operation, a para-stomal hernia was found in the patient, but after conservative treatment, the posterior hernia sac gradually enlarged, and more mesentery entered the abdominal wall through the fistula to form a fistula hernia. Obvious expansion and effusion could be found in the intestinal canal, stomach lumen and esophagus, and there were multiple gas-liquid planes, so intestinal obstruction was considered. In October 2019, the patient underwent para-stomal hernia repair, sigmoidostomy repair and ileostomy, and peritoneal nodules biopsy was performed at the same time. Postoperative pathology showed metastatic adenomas in the right umbilical fold ligament, left iliac fossa and pelvic. According to the results of chemotherapy and clinical manifestations, we believed that the patient was not tolerant to oxaliplatin, so we decided to change the chemotherapy regimen instead of XELOX.

### Second-line treatment

The molecular typing of the patient was UGT1A1 (+). Because irinotecan was effective in the patients with UGT1A1 positive, we replaced the chemotherapy regimen with FORFIRI (irinotecan 200mg/m^2^, calcium folinate 100mg/m^2^ and 5-fluorouracil 400mg/m^2^) and bevacizumab(5mg/Kg) for 12 cycles from October 2019 to December 2020. In the course of this chemotherapy, the patients were resistant to chemotherapeutic drugs and few adverse reactions happened. The re-examination of CT showed that the size and number of bilateral pulmonary nodules were the same as before. Unfortunately, the low-density shadows within the liver increased and enlarged. In December 2020, the result of PET-CT suggested that the tumor at the anastomotic recurred with multiple metastases.

### Third-line treatment and immune-related keratitis

**Figure 2:**
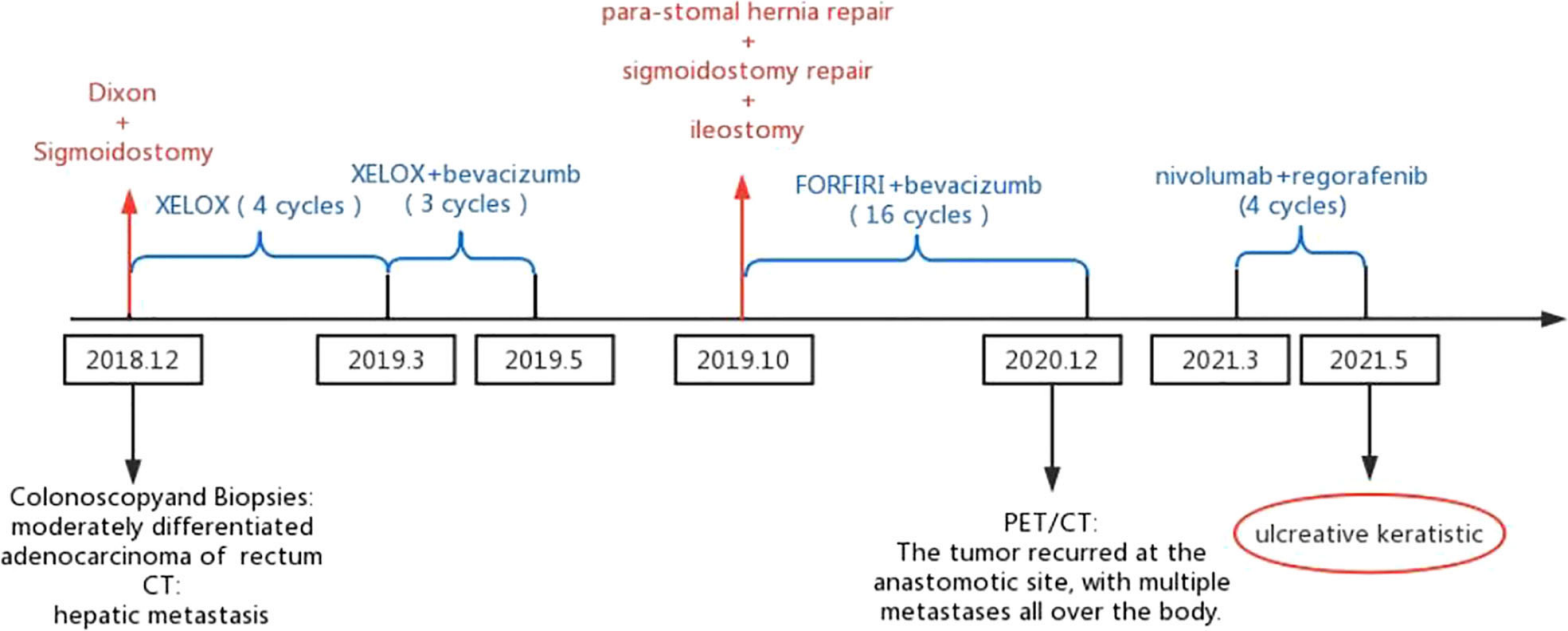
The patient underwent two surgeries (Dixon and sigmoidostomy conducted in December 2018 and para-stomal hernia repair, sigmoidostomy repair and ileostomy conducted in October 2019). The patient received 28 chemotherapies (XELOX for sessions 1-4, XELOX plus bevacizumab for sessions 5-7, FORFIRI plus bevacizumab for sessions 8-23, nivolumab plus regorafenib for sessions 24-28).

Combined with the pathological stage and the wishes of the family, We gave the patient palliative treatment. According to the CSCO guidelines, we chose to use nivolumab plus regorafenib as the third-line chemotherapy drug for this patient. After treatment with nivolumab, the effect was better, and the CEA value decreased gradually. So far, the patient underwent 2 operations and 28 times of chemotherapy (**Figure 2**).

**Figure 3:**
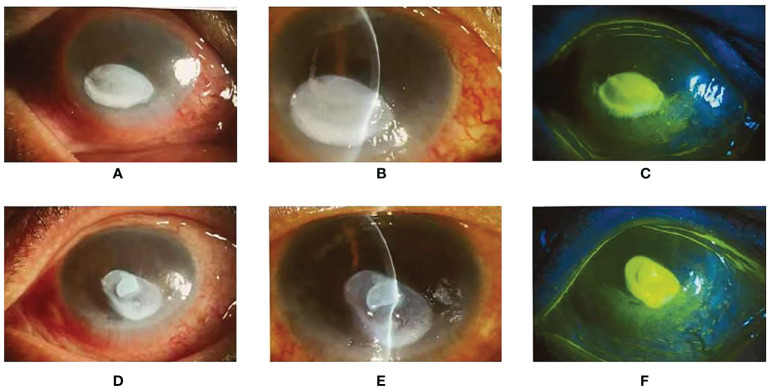
**(A, B)**: The right eye slit-lamp examination and anterior segment photography. There are mixed eyelid congestion and oval ulceration (3*5mm) in the central cornea. The opacity of the corneal lesions, edema around the lesions, and mild opacity of the other peripheral cornea can be observed. **(D, E)**: The left eye slit-lamp examination and anterior segment photography. The size of the ulceration is 3*6mm, other characteristics are the same as the right eye. **(C, F)**: Lamellar staining of the corneal epithelium in the ulceration of both eyes with fluorescent, and punctate staining and positive seidel test were seen in the rest of the cornea.

After the 28th chemotherapy, the patient came to the ophthalmology clinic of our hospital because of photophobia and tearing in both eyes with decreased vision. The patient was admitted for further evaluation, and a retrospective history revealed that on the fourth day after the completion of the fourth course of nivolumab plus regorafenib, the patient developed photophobia, tearing, and increased white secretions in both eyes. Besides, the patient had significant visual impairment: Vod:0.3(corrected:0.4), Vos:0.5, IOP of the right eye: 16mmHg, IOP of the left eye: 17mmHg.The anterior chamber of both eyes had normal depth and clear aqueous humor. Slit-lamp examination and anterior segment photography showed evident ulceration in both eyes, and fluorescent(FL) showed lamellar corneal epithelial staining and a positive seidel test (**Figure 3**). Combined with other examination results, this patient was diagnosed with corneal ulceration in both eyes and admitted to the ophthalmology ward. Considering that the patient still needed to treat colon cancer, he was transferred back to the general surgical ward.

Then we diagnosed the cause of corneal ulceration. First of all, since infectious keratitis is one of the most common causes of corneal ulceration, we gave priority to judging whether the patient had infectious keratitis. Corneal scraping samples in both eyes were taken for microbiological examination, such as Gram staining, bacterial culture and fungal culture, and the results were all negative. In addition, the symptoms of the patients were not significantly improved after empirical anti-infective treatment.

During the treatment, the patient self-reported dry mouth, joint pains and low tear secretion, SchirmerI = 0mm/5min. Quantitative autoantibody testing was performed on this patient after consultation with a rheumatology specialist. All the test reports were negative, and the quantitative results were far less than the reference values.

Next, we ruled out the possibility of neurotrophic keratitis. The patient received symptomatic treatment, including neurotrophic treatment, repair promotion, and anti-infective treatment. However, the effect was not evident after the use of artificial tears, Diquafosol Sodium Eye Drops, Recombinant Bovine Basic Fibroblast Growth Factor Eye Gel and Eye Drops, and Levofloxacin Eye Drops.

**Figure 4:**
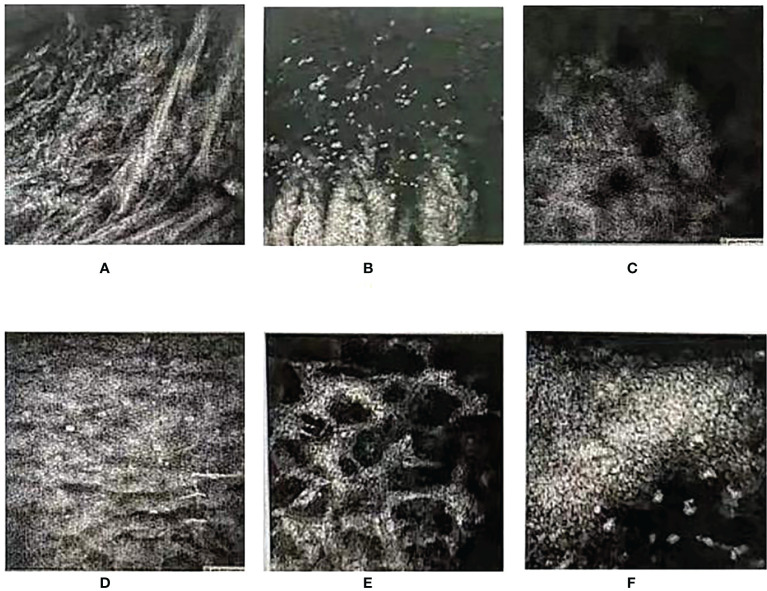
In-vivo histology of the cornea (zeiss63X, FOV400μm). **(A, B)** Below the cornea of the left eye, the epithelial layer of the lesion was found to be defective. Many streak-like hyper-reflections and no normal epithelial cells were observed. **(C, D)** Deep structures were not imaged. Epithelial cells of the corneal epithelium in the clear zone above the lesion were swollen, and subepithelial nerve fibers were not observed. **(E, F)** The superficial stromal layer was slightly turbid, and the deep stromal cells were activated. The shape of endothelial cells was good, and there were a large number of punctate hyper-reflections observed in the endothelial layer.

Therefore, we highly suspected that the cause of the corneal ulceration of this patient was the immune-related adverse reactions of the PD-1 monoclonal antibody. Based on the previous medication, glucocorticoids and autologous serum were used as a diagnostic treatment, which achieved a good effect. Due to adverse ocular reactions, we temporarily suspended chemotherapy for this patient. One month later, the re-examination showed that the eye condition of the patient recovered well, so the fifth cycle of chemotherapy with nivolumab and regorafenib. However, after the chemotherapy, the patient’s ocular symptoms were significantly aggravated (VOD: 0.075, VOS: 0.0125), and the anterior chambers of both eyes disappeared. The slit-lamp examination and photography of the anterior eye segment showed ulceration in both eyes, and the seidel test was positive. *In-vivo* histology of the cornea suggested that the patient developed corneal ulceration with perforation (**Figure 4**). After excluding the relevant surgical contraindications, the surgery was performed: the patient underwent multi-layered amniotic membrane filling in the left eye, and multi-layered amniotic membrane filling, anterior chamber paracentesis as well as peripheral iridectomy in the right eye. There was continuous use of Tobramycin and Dexamethasone Eye Drop postoperatively. The postoperative re-examination revealed the eutopic AM, the transparent remaining cornea, FL (–), and keratic precipitate (–) in both eyes. Because of severe corneal ulceration after re-administration of nivolumab, we believed that nivolumab was responsible for the patient’s immune-related keratitis.

## Discussion

### Diagnosis and treatment basis of immune-related keratitis

The patient has advanced colorectal cancer with liver metastases. After completing the fourth cycle of nivolumab combined with regorafenib, the patient began to develop symptoms such as binocular photophobia, tearing, decreased vision and eventually developed corneal ulceration. We excluded infectious keratitis because the microbial cultures were negative and anti-infective treatment alone was ineffective. During the treatment, the patient self-reported dry mouth, dry eyes, and joint pain in the extremities, and because the most common comorbidity of rheumatoid arthritis is dry eyes, which was consistent with the patient’s clinical presentation. We suspected that the patient had rheumatoid arthritis combined with dry eyes, but the diagnosis was ruled out after checking for autoantibodies. Considering patients who received treatments that inhibit the PD-1 and PD-L1 pathways may present with optic neuritis and hypopituitarism (10), We suspected neurotrophic keratitis (NK), a rare degenerative disease with impaired epithelial healing (11–13). In particular, most adults with moderate or severe NK have complete corneal healing and a low rate of disease progression after up to 8 weeks of topical treatment (14). However, the patient did not show significant improvement after treatment with Recombinant Bovine Basic Fibroblast Growth Factor Eye-Gel.

After literature review, combined with the patient’s onset time, the initial diagnosis was irAE keratitis, similar to peripheral ulcerative keratitis (9). Since corneal matrix metalloproteinases (MMPs) are a contributing factor to corneal ulceration, glucocorticoids can effectively inhibit the activity of MMPs and have immunosuppressive effects (8, 15).MMPs inhibitors in autologous serum can effectively inhibit the activity of MMPs, globulin in serum inhibit the activity of corneal collagenase, and fibronectin promotes epithelial cell migration and adhesion, which have a good effect on the prevention of corneal perforation (16–18). Therefore, based on the original treatment, we added glucocorticoid combined with autologous serum for diagnostic treatment. After treatment, the patient’s symptoms improved significantly, which suggested that this was immune-related keratitis.

Currently, according to the recommended guidelines for irAE management, ocular-related irAE at grade III should be temporarily discontinued until resolution. And the treatment should be permanently discontinued (19, 20) at Grade IV (Vision is worse than 20/200). Due to adverse ocular reactions and visual acuity of Vod:0.3 (corrected:0.4) and Vos:0.5, the chemotherapy was suspended. After one month, a review showed that the patient’s eyes were recovering well, and we proceeded to the fifth cycle of nivolumab combined with regorafenib. However, after chemotherapy the patient again developed corneal ulceration and the patient’s visual acuity (VOD:0.075, VOS:0.0125) was irAE grade IV. According to the guidelines, nivolumab therapy was stopped. Therefore, we confirmed that the patient developed a corneal ulcer as a result of treatment with nivolumab in combination with regorafenib.

Two cases of corneal ulceration following nivolumab treatment were reported. Jack S. Parker reported a woman who developed a recalcitrant corneal ulcer after one month of nivolumab treatment (21). Gergely Losonczy reported a man who developed severe symptoms of dry eyes and persistent corneal epithelial defects after nivolumab treatment. They found that the levels of inflammatory cytokines and MMPs in tears were significantly increased after nivolumab treatment (22). However, these two cases are both about the treatment of melanoma, and no relevant case report has been found regarding patients with advanced colorectal cancer who developed corneal ulcers after making nivolumab treatment.

### Possible mechanism

Although this is a rare complication, the case demonstrates that immune-related keratitis can occur in mCRC patients treated with nivolumab. However, since the understanding of immune-related keratitis mainly comes from a few case reports, besides, there are fewer reports in patients with advanced metastatic colorectal cancer mCRC treated with nivolumab, so we cannot summarize the specific clinical manifestations of the disease. Therefore, the diagnosis of the disease often brings a lot of trouble to clinicians. In this regard, we refer to a large number of papers to explain the causes of immune-related keratitis caused by nivolumab from the following possible mechanisms.

In previous studies, we have known that the eye is an immune-privileged site, which depends on the anatomy of the eye and the immune microenvironment (23, 24). The first is the blood-eye barrier, which limits the entry of hematogenous immune effector cells and molecules; the second is that upregulation of Fas ligand (Fas-L) and tumor growth factor (TGF-β) in the ocular immune microenvironment can convert T cells to Treg, reconstituting the immunosuppressive microenvironment and anterior chamber-associated immune deviation (ACAID), and help the eye avoid autoimmune diseases (9, 24). In addition, there is one more mechanism that can overcome the intraocular immune response dominated by Th1 and reconstruct ACAID, although it is not yet fully understood (24). Some studies have shown that PD-L1 expressed by corneal endothelial cells can bind to PD-1, thereby inhibiting the function of Th1 (9). It is reasonable to suspect that nivolumab blocks the binding of PD-1 to PD-L1 expressed by corneal endothelial cells. That is, After nivolumab was used, PD-L1 and PD-1 pathway was suppressed, so Th1 function could not be inhibited. Moreover, ACAID is characterized by inhibition of antigen delayed type hypersensitivity (DTH) and loss of complement binding antibodies (25). Studies have shown that immunosuppressive drugs can inhibit the production of regulatory T cells (Treg). Two types of Treg are produced during the formation of ACAID, one is CD4+Treg, which inhibits DTH response, and the other is CD8+Treg, which inhibits DTH response (25–28). To sum up, nivolumab weakens the inhibitory effect of the corneal microenvironment on th1 and inhibits the production of Treg, collectively, leading to disruption of the immune microenvironment and ACAID.

Besides, nivolumab may affect the ability of corneal healing. In one study, researchers identified maspin in the corneas of horses with immune-related keratitis(IMMK), which is a tumor suppressor protein that regulates cell migration, invasion and adhesion and is synthesized in many normal epithelial cells. They suggested that the etiology of IMMK in horses may be an autoimmune response, and maspin plays a role in it (29, 30). Maspin has been identified in the human corneal epithelium, stroma and endothelium. When the cornea is injured, the stromal keratinocytes below the wound undergo apoptosis and their neighbors transform into fibroblasts or myofibroblasts. In this process, maspin acts directly on endothelial cells to stop their migration towards basic fibroblast growth factor(bFGF) and vascular endothelial growth factor(VEGF) (31–33). Therefore, we speculate that maspin may affect the healing of the injured cornea. Other studies have found that when maspin is overexpressed *in vivo* targeting endothelial cells, it will actively induce endothelial cell apoptosis, and neovascularization endothelial cells are highly sensitive to the level of intracellular maspin (34). As there have been previous reports of autoimmune diseases in patients using nivolumab, we believe that nivolumab will lead to the occurrence of autoimmune diseases (35–37). For eyes, maspin interferes with corneal healing may be the cause of immune-related keratitis caused by nivolumab. As previously mentioned, Gergely Losonczy found that patients with immune-related keratitis had elevated levels of MMPs, which contribute to the formation of corneal ulceration (22, 38). Unfortunately, we did not detect MMPs and maspin in the patient. However, we reasonably speculate that MMPs and maspin are the common promoting factors leading to immune keratitis. The detection of MMPs and MSAPIN in patients with nivolumab may be a method to prevent and diagnose corneal irAE.

### Relationship to treatment outcome

The current research shows a universal association between irAE and patient reactivity with anti-PD-1 antibodies. In particular, endocrine, dermatologic and low-grade irAEs are associated with better ICI efficacy (39). Patients who experienced irAE had better survival than those who did not (40). However, it was not identified how the severity of the irAE, the site of onset and the treatment affected the effect of ICI (41). And the relationship between the occurrence of ocular irAEs and the efficacy of ICI has not been researched. After the patient was treated with nivolumab, despite concurrent ocular irAE, CEA showed a surprising and gradual decline. This suggests that ocular irAE may be associated with better ICI efficacy. In subsequent research, this case can be used to research the relationship between irAE and ICI efficacy.

## Conclusion

We report a case of immune keratitis with perforation caused by third-line immunotherapy for advanced intestinal cancer of the MSS staging. It is speculated that nivolumab damages the immune microenvironment and ACAID and interferes with the ability to heal corneal injury, which is the mechanism of immune-related keratitis. Monitoring of MMPs and MSAPIN in patients treated with nivolumab may be a method to prevent and diagnose immune keratitis. Immune-related keratitis may be considered after infectious and autoimmune etiologies have been excluded. This type of keratitis is highly effective while the combination of glucocorticoids and autologous serum, which can be used for diagnostic treatment. In addition. The occurrence of immune keratitis may be an indicator of the efficacy of ICI, which can be further investigated in the follow-up. As this is only a case report, the mechanism of immune keratitis and the relationship with the efficacy of ICI are still to be investigated. In subsequent studies, this case can be used as reference material to enable more scholars to have further research on the diagnosis and treatment of immune keratitis.

## Data availability statement

The original contributions presented in the study are included in the article/supplementary material. Further inquiries can be directed to the corresponding authors.

## Ethics statement

Written informed consent was obtained from the individual(s) for the publication of any potentially identifiable images or data included in this article.

## Author contributions

YS: literature research, manuscript preparation. JiZ: literature research, manuscript preparation. JX: literature research, manuscript preparation. JaZ: literature research. XG: literature research. ZC: manuscript final version approval. ZL: manuscript final version approval. SH: manuscript final version approval. All authors contributed to the article and approved the submitted version.

## Funding

This work was supported by Special Funds for the Cultivation of Guangdong College Students’ Scientific and Technological Innovation (Grant No. pdjh2021a0094) and Support Program for Chinese Youth Innovation and Entrepreneurship Project (Grant No. PDJH2021008). The foundations play a role in encouraging college students to take an active part in scientific and technological innovation. The authors declare that all sources of funding received for the research being submitted and the funds are received for open access publication fees from the above grant.

## Conflict of interest

The authors declare that the research was conducted in the absence of any commercial or financial relationships that could be construed as a potential conflict of interest.

## Publisher’s note

All claims expressed in this article are solely those of the authors and do not necessarily represent those of their affiliated organizations, or those of the publisher, the editors and the reviewers. Any product that may be evaluated in this article, or claim that may be made by its manufacturer, is not guaranteed or endorsed by the publisher.
